# Patients with Type 2 Diabetes Initiating Exenatide Twice Daily or Insulin in Clinical Practice: CHOICE Study

**DOI:** 10.1007/s13300-012-0006-7

**Published:** 2012-06-20

**Authors:** Stephan Matthaei, Matthew Reaney, Chantal Mathieu, Claes-Göran Östenson, Thure Krarup, Bruno Guerci, Jacek Kiljanski, Helmut Petto, David Bruhn, Michael Theodorakis

**Affiliations:** 1 Diabetes-Zentrum Quakenbrück, Fachabteilung fur Diabetologie, Stoffwechsel und Endokrinologie am Christlichen Krankenhaus, Klinisches Diabeteszentrum der DDG, Akademisches Lehrkrankenhausder Medizinischen Hochschule Hannover, Danziger Str.10, 49610 Quakenbruck, Germany; 2 Eli Lilly, Windlesham, Surrey, UK; 3 Department of Endocrinology, UZ Gasthuisberg, Leuven, Belgium; 4 Department of Molecular Medicine and Surgery, Karolinska Institutet, Stockholm, Sweden; 5 Department of Endocrinology I, Bispebjerg Hospital, Copenhagen, Denmark; 6 Diabetology, Nutrition, Metabolic Disorders, Brabois Hospital and Center of Clinical Investigation ILCV, CHU de Nancy, Vandoeuvre-Les-Nancy, France; 7 Eli Lilly, Warsaw, Poland; 8 Eli Lilly, Vienna, Austria; 9 Eli Lilly, San Diego, CA USA; 10 Department of Clinical Therapeutics, University of Athens School of Medicine, Athens, Greece

**Keywords:** Exenatide, Insulin, Type 2 diabetes mellitus

## Abstract

**Introduction:**

Changes to Treatment and Outcomes in Patients with Type 2 Diabetes Initiating Injectable Therapy (CHOICE) is a European prospective, observational cohort study assessing time to, and factors associated with, a significant change in therapy after type 2 diabetes patients initiate their first injectable glucose-lowering therapy, and these patients’ clinical outcomes over 24 months. The authors report baseline data and factors associated with the injectable treatment regimen.

**Methods:**

Demographic, clinical, and healthcare resource-use data were collected at initiation of injectable therapy and analyzed using univariate tests between cohorts and multivariate logistic regression analysis for treatment.

**Results:**

Overall, 1,177 patients initiated exenatide twice daily (b.i.d.) and 1,315 initiated insulin. Most patients were recruited by secondary-care physicians. Univariate analyses revealed statistically significant differences between the characteristics of patients who initiated exenatide b.i.d. and patients who initiated insulin. On multivariate analysis, higher body mass index [BMI; 5 kg/m^2^ higher: odds ratio (OR) 2.10, 95% confidence intervals (CI) 1.84–2.40], lower glycated hemoglobin (HbA_1c_; 1% higher: OR 0.77, 95% CI 0.69–0.86), and lower age (5 years older: OR 0.82, 95% CI 0.76–0.88) were the variables most strongly associated with increased probability of receiving exenatide b.i.d. (*P* < 0.0001). Patients initiating exenatide b.i.d. had a mean BMI of 35.3 ± 6.5 kg/m^2^, HbA_1c_ of 8.4 ± 1.4%, and age of 58 ± 10 years, compared with 29.7 ± 5.4 kg/m^2^, 9.2 ± 1.9%, and 64 ± 11 years, respectively, in patients initiating insulin (*P* < 0.0001). Other characteristics significantly associated with exenatide b.i.d. initiation were “disinhibited eating” (Diabetes Health Profile-18), lower random blood glucose, less blood glucose self-monitoring, lower low-density lipoprotein cholesterol, and receipt of diet/exercise advice.

**Conclusions:**

Patients who initiated exenatide b.i.d. were on average younger and more obese with lower HbA_1c_ than those initiating insulin.

## Introduction

During the enrollment period for the CHanges to Treatment and Outcomes in Patients with Type 2 Diabetes Initiating Injectable Therapy (CHOICE) study, exenatide twice daily (b.i.d.), the first approved glucagon-like peptide-1 (GLP-1) receptor agonist, was available in Europe for use in combination with metformin and/or a sulfonylurea in patients with type 2 diabetes mellitus (T2DM) with insufficient glycemic control on maximal doses of these medications. Indications for exenatide b.i.d. in Europe have subsequently been expanded to include use in combination with thiazolidinediones (in 2010) and as adjuvant therapy with basal insulin, with or without metformin and/or pioglitazone, in patients who had not achieved adequate glycemic control with these agents (in 2012). In head-to-head phase 3 clinical trials, exenatide b.i.d. and insulin (glargine and biphasic insulin aspart) provided similar glycemic control in patients whose diabetes was not controlled with oral antidiabetic medications (OADs). Exenatide b.i.d. treatment was associated with weight loss, while patients randomized to insulin typically gained weight [1–3]. Metabolic improvements with exenatide b.i.d. were maintained in a subset of patients treated for 3.5 years [4].

While randomized, controlled trials are the reference standard for assessing the efficacy and safety of therapy, large, observational studies are necessary to determine how glucose-lowering medications are used in clinical practice and to evaluate their effectiveness and safety in this setting [5, 6]. The patterns of exenatide b.i.d. usage in clinical practice across Europe have not previously been evaluated. For example, it is unclear which patients requiring injectable glucose-lowering therapy are initiated on exenatide b.i.d. A comparison of the baseline characteristics of patients initiating exenatide b.i.d. and insulin is, therefore, of interest and could indicate whether the selection of injectable therapy is in accordance with known clinical differentiation and published guidelines [7]. Other gaps in knowledge include how, when, and why treatment is intensified or switched (e.g., by the addition of other glucose-lowering medications or by the substitution of one insulin regimen with another) and the clinical response in routine care. Primary-care databases, the principal source of retrospective observational data, are of limited use in this regard because exenatide b.i.d. is commonly (although not exclusively) initiated in secondary care.

CHOICE is the first European observational study conducted specifically to address this lack of evidence. CHOICE is an ongoing, prospective cohort study designed to assess the time to a significant subsequent change in therapy among patients who initiate their first injectable glucose-lowering therapy with either exenatide b.i.d. or insulin (the only injectable treatments available when this study commenced) in Europe. The study also aimed to describe the characteristics of patients with T2DM initiating injectable therapy, factors associated with treatment changes, and clinical outcomes and common adverse events observed over 24 months. CHOICE is being conducted in six European countries and will be completed in 2012. This paper reports the baseline characteristics of enrolled patients and measured variables associated with injectable treatment regimen.

## Methods

### Design and Patients

CHOICE is a prospective, multinational, non-interventional observational study being conducted in Belgium (31 sites), Denmark (eight sites), France (71 sites), Germany (130 sites), Greece (49 sites), and Sweden (33 sites). The primary endpoint is time spent on initial injectable regimen (exenatide b.i.d. or insulin) before significant treatment change. Significant treatment change is defined as at least one of the following (and does not include switching between brands of the same class/type of insulin): addition of a new medication (any route of administration) for treatment of T2DM; a change in the number of times insulin is administered per day; discontinuation of any exenatide b.i.d./insulin initiated at baseline; substitution of a human insulin for an analog insulin or vice versa.

Secondary objectives include assessment of characteristics of patients initiating each treatment, factors associated with injectable treatment regimen, and clinical and patient-reported outcomes.

Eligible for inclusion were adults aged ≥18 years initiating their first injectable glucose-lowering therapy with exenatide b.i.d. or insulin for the treatment of T2DM in routine clinical practice. At study entry patients could be taking any OADs. Patients were initiated on either exenatide b.i.d. or insulin according to clinical decision making, and were then informed about CHOICE and invited to participate. Patients gave written informed consent for the use of their data. Appropriate ethical review board approval was obtained for this study.

Patients have been assessed at study visits at the time of initiation of injectable therapy (baseline, reported here) and approximately 3, 6, 12, 18, and 24 months thereafter, as per routine care. Patients referred from the study site to another healthcare provider during the study have been followed up by contacting, and obtaining the consent of, the new provider and by postal patient questionnaires. Interim analyses are planned at baseline, 6, and 12 months, with the final analysis after the 24-month visit.

### Data Collected

At baseline (initiation of injectable therapy), standard clinical data were collected from each patient, i.e.,: demographic characteristics; clinical characteristics (current and historical), including glycated hemoglobin (HbA_1c_; at initiation and over previous 2 years), weight, blood pressure, lipid levels, and diabetes complications; retrospectively recalled incidence of self-reported hypoglycemic episodes (over the preceding 3 months) and gastrointestinal symptoms (over the preceding 4 weeks); previous and ongoing diabetes therapy and care; concomitant medications; and patient-reported outcome measures of health status and functioning, including the Diabetes Health Profile (DHP)-18 instrument [8]. Data were collected using an electronic data capture form: clinical data were entered by the investigator (or proxy); patient-reported outcome data were provided by the patient and transferred to the same form by the site personnel.

### Analysis

#### Sample Size Justification

The study aimed to recruit a maximum of 800 patients per country/country group with a ratio of approximately 60% initiating insulin and 40% initiating exenatide b.i.d. The sample size calculation was performed by Monte-Carlo simulation assuming patient drop-out rates of 10–15% per year and median time to significant treatment change of 9.0 months for the exenatide b.i.d. cohort and 8.6 months for the insulin cohort [9, 10]. The insulin cohort was larger than the exenatide b.i.d. cohort owing to greater variability in the former (linked to use of different insulin regimens), which necessitated a larger population in order to achieve similar precision for the estimation of time to treatment change [95% confidence intervals (CI) of 3–4 months width around median within countries and cohorts].

The following strategy was used to achieve the required number of patients in each cohort without intervening in treatment decisions made during normal clinical practice: once a cohort within a participating country was filled, investigating physicians were asked to stop enrollment into that cohort and to continue enrolling patients only into the other treatment cohort, as and when they initiate patients on that treatment according to their usual practice.

#### Statistical Analysis

All patients eligible at baseline were included in the analyses. Baseline patient data were reported using descriptive statistics and 95% CI where appropriate. For continuous variables mean, SD, median, minimum, maximum, and quartiles were calculated. Absolute numbers and percentages (including missing values) were given for categorical variables. Per-country analyses were also performed.

Univariate analyses were performed to compare all baseline patient characteristics between the two cohorts (overall population and per country). Continuous variables were analyzed using *t* tests, analyses of variance (ANOVA), or where necessary the corresponding nonparametric alternatives (e.g., Wilcoxon signed rank test). Categorical variables were analyzed using χ^2^ tests, Fisher’s exact tests, and trend tests. Logistic regression models were then applied to identify factors significantly associated with injectable treatment regimen (differentiation between exenatide b.i.d. and insulin), using forward selection processes and including only those variables that were statistically significant (*P* < 0.1) at the univariate level. For all these analyses missing data were not imputed.

Logistic regression was also used to derive propensity scores from baseline data (0.10 threshold for between-cohort differences). The propensity score estimates the probability that a patient will be assigned to a treatment group based on baseline characteristics (score range 0–1). Propensity score analysis was used to assess comparability of the treatment cohorts [11]. For this analysis all eligible baseline data were included. Missing data were imputed with the overall mean or median for continuous variables, and most frequent category for categorical variables, in order to give a conservative estimate. Patients were matched 1:1 by country based on the propensity score and optimal matching to identify matched subsets from the two cohorts. All *P* values are reported without multiplicity adjustments.

## Results

Between January 2008 and October 2009, 2,513 patients were recruited; 2,492 were eligible for inclusion in CHOICE. Overall, 1,177 (47.2%) patients initiated exenatide b.i.d. and 1,315 (52.8%) initiated insulin. Almost half (46%) of patients initiating insulin received basal-only insulin, 23% received mixtures, 13% basal-bolus regimen, 11% short-acting only, and 7% other or missing, although there was significant between-country variability. Numbers of participants in each country were: Belgium, 299 (43.1% exenatide b.i.d.); Denmark, 60 (73.3% exenatide b.i.d.); France, 290 (67.6% exenatide b.i.d.); Germany, 848 (46.5% exenatide b.i.d.); Greece, 807 (39.4% exenatide b.i.d.), and Sweden, 188 (51.1% exenatide b.i.d.). Of the 325 investigators, 220 (67.7%) were secondary-care physicians and 23 (7.1%) were primary-care physicians [“other” or missing data: 82 (25.2%)].

### Demographic and Clinical Characteristics

**Fig. 1:**
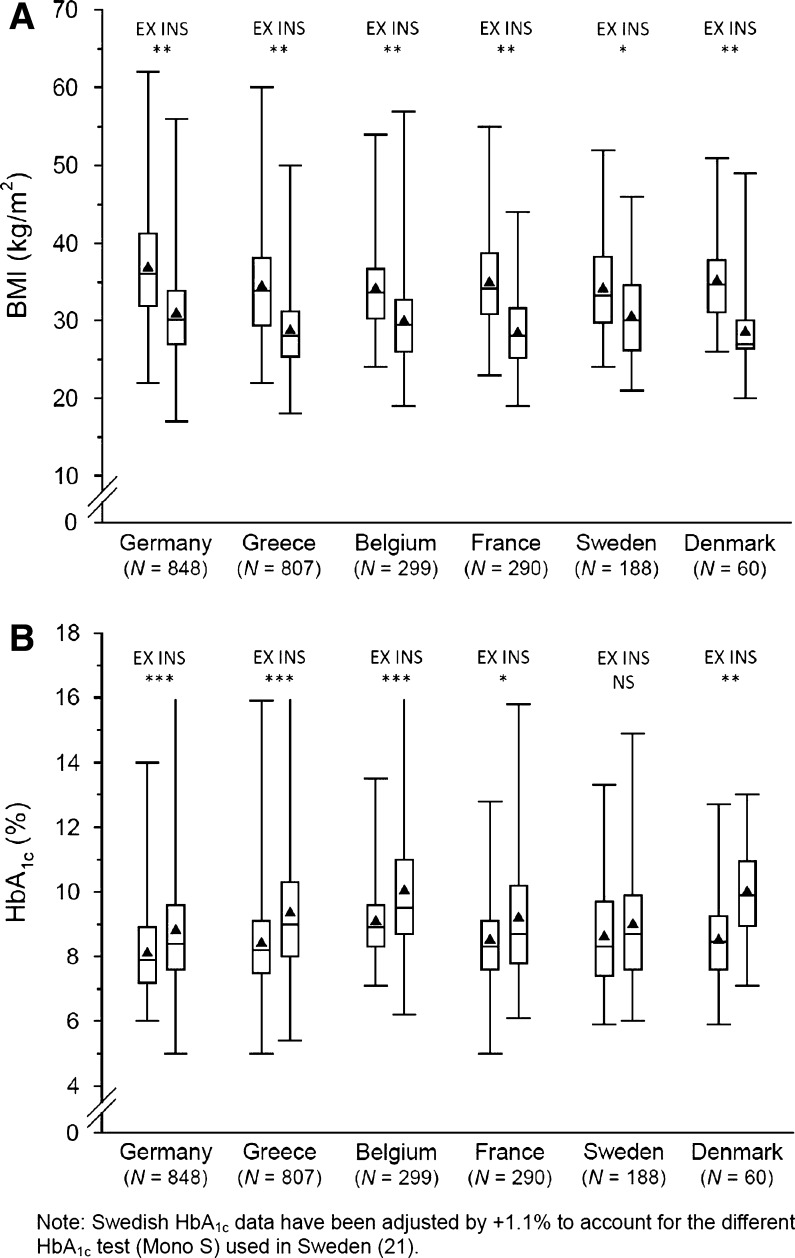
BMI (**a**) and HbA_1c_ values (**b**) (most recent during past 3 months) among patients with type 2 diabetes at the initiation of exenatide b.i.d. (EX) or insulin (INS) in six countries. *Box plots* show mean (*triangle*), median (*line*), 25% and 75% quartiles (*box*) and minimum/maximum values (*whiskers*). **a** *P < 0.001, **P < 0.0001 for intercohort comparison (Wilcoxon text), **b** *P = 0.01, **P = 0.001, ***P < 0.0001 for intercohort comparison (Wilcoxon text). *b.i.d.* twice daily, *BMI* body mass index, *HbA*_*1c*_ glycated hemoglobin, *NS* not significant

Overall, patients had a mean age of 61 ± 10 years, BMI of 32.3 ± 6.6 kg/m^2^ and HbA_1c_ of 8.9 ± 1.7%. Mean duration of diagnosed diabetes was 9 ± 7 years. Univariate analyses revealed statistically significant differences between patients whom clinicians initiated on exenatide b.i.d. and starter insulin regimens (collectively referred to as “insulin”; Table 1). Patients initiating exenatide b.i.d. were on average significantly younger than those initiating insulin (mean age 58 ± 10 vs. 64 ± 11 years; *P* < 0.0001). This trend was consistent across all countries except Denmark, wherein the cohorts did not differ significantly in age (total *n* = 60). Patients initiating exenatide b.i.d. had significantly higher mean body weight (101.1 ± 21.6 vs. 84.3 ± 17.6 kg; *P* < 0.0001) and mean BMI (35.3 ± 6.5 vs. 29.7 ± 5.4 kg/m^2^; *P* < 0.0001) than those initiating insulin (Table 1), a finding that was also consistent across all countries (Fig. 1a). Exenatide b.i.d. patients also had a higher mean waist circumference in all countries (nonsignificant difference in Denmark). Exenatide b.i.d. patients had higher mean diastolic blood pressure and lower mean total cholesterol, low-density lipoprotein cholesterol, and creatinine values. The two cohorts also differed significantly in educational status when all subcategories of educational level were taken into account (Table 1), although data were available for only 1,937 patients (77.7% of total) and hence this was not included in the multivariate analysis.

**Table 1:** Baseline characteristics of patients initiating exenatide b.i.d. or insulin

Variable	Exenatide b.i.d. (*n* = 1,177)	Starter insulin (*n* = 1,315)	*P* value^a^	Total (*n* = 2,492)
Male, *n* (%)	635 (54.0)	762 (57.9)	0.0427	1,397 (56.1)
Caucasian, *n* (%)	970 (82.4)	1,206 (91.7)	NS	2,176 (87.3)
Age, years	58 ± 10	64 ± 11	<0.0001	61 ± 11
Weight, kg	101.1 ± 21.6	84.3 ± 17.6	<0.0001	92.2 ± 21.3
BMI, kg/m^2^	35.3 ± 6.5	29.7 ± 5.4	<0.0001	32.3 ± 6.6
Waist circumference, cm	114.6 ± 14.8	103.3 ± 14.1	<0.0001	108.7 ± 15.5
Blood pressure, mmHg				
Systolic	137.8 ± 16.5	137.4 ± 17.4	NS	137.6 ± 17.0
Diastolic	81.6 ± 9.6	80.1 ± 9.9	<0.0001	80.8 ± 9.8
Plasma lipids, mmol/L^b^				
Total cholesterol	4.93 ± 1.06	5.12 ± 1.23	0.0007	5.03 ± 1.15
LDL cholesterol	2.82 ± 1.09	3.00 ± 1.01	<0.0001	2.91 ± 1.05
HDL cholesterol	1.18 ± 0.34	1.19 ± 0.34	NS	1.19 ± 0.34
Triglycerides	2.37 ± 1.54	2.36 ± 1.99	0.0212	2.36 ± 1.79
Creatinine, mmol/L^b^	82.6 ± 40.1	91.1 ± 59.1	<0.0001	87.1 ± 51.3
Smoking status, *n* (%)				
Ever smoked	498 (42.3)	512 (38.9)	0.0307	1,010 (40.5)
Current smoker	178 (15.1)	229 (17.4)	NS	407 (16.3)
Employment, *n* (%)			<0.0001^c^	
Working full/part time	483 (41.0)	356 (27.1)		839 (33.7)
Retired	402 (34.2)	681 (51.8)		1,083 (43.5)
Unemployed and other	292 (24.8)	278 (21.1)		570 (22.9)
Education, *n* (%)			<0.0001^c^	
No formal	69 (5.9)	71 (5.4)		140 (5.6)
Minimum mandatory	437 (37.1)	572 (43.5)		1,009 (40.5)
Further education	310 (26.3)	245 (18.6)		555 (22.3)
University	127 (10.8)	106 (8.1)		233 (9.3)
Unknown	229 (19.5)	315 (24.0)		544 (21.8)
Comorbid illness, *n* (%)			0.0014^d^	
Hypertension	818 (69.5)	859 (65.3)		1,677 (67.3)
Hyperlipidemia	642 (54.5)	641 (48.7)		1,283 (51.5)
Concomitant therapy, *n* (%)				
Any	1,016 (86.3)	1,114 (84.7)	NS	2,130 (85.5)
Lipid-lowering	664 (56.4)	712 (54.1)	NS	1,376 (55.2)
Cardiovascular	895 (76.0)	972 (73.9)	NS	1,867 (74.9)
Antiplatelet	485 (41.2)	599 (45.6)	NS	1,084 (43.5)
Weight-lowering	54 (4.6)	20 (1.5)	<0.0001	74 (3.0)
Time since diabetes diagnosis, years	8 ± 6	10 ± 7	<0.0001	9 ± 7
HbA_1c_, most recent in previous 3 months, %	8.4 ± 1.4	9.2 ± 1.9	<0.0001	8.9 ± 1.7
HbA_1c_ <7%, *n* (%)	126 (10.7)	74 (5.6)	–	200 (8.0)
Random blood glucose, mmol/L	10.4 ± 3.1	12.1 ± 4.4	<0.0001	11.4 ± 4.0
Use of SMBG, *n* (%)	928 (78.8)	1,050 (79.8)	NS	1,978 (79.4)
No. of test strips used (per week)^e^	9.3 ± 7.9	9.9 ± 8.6	NS	9.6 ± 8.3
No. of OADs used (previous 12 months), *n* (%)				
0	197 (16.7)	173 (13.2)	–	370 (14.8)
1	379 (32.2)	445 (33.8)	–	824 (33.1)
2	467 (39.7)	521 (39.6)	–	988 (39.6)
≥3	134 (11.4)	176 (13.4)	–	310 (12.4)
Antidiabetic medication class used (previous 12 months), *n* (%)				
Αlpha-glucosidase inhibitor	15 (1.3)	21 (1.6)	–	36 (1.4)
Biguanide	816 (69.3)	881 (67.0)	–	1,697 (68.1)
Biguanide + sulfonylurea	33 (2.8)	39 (3.0)	–	72 (2.9)
DPP-4 inhibitor	81 (6.9)	97 (7.4)	–	178 (7.1)
GLP-1 receptor agonist	2 (0.2)	0 (0.0)	–	2 (0.1)
Secretion enhancer	75 (6.4)	99 (7.5)	–	174 (7.0)
Sulfonylurea	494 (42.0)	682 (51.9)	–	1,176 (47.2)
Thiazolidinedione	136 (11.6)	150 (11.4)	–	286 (11.5)
Thiazolidinedione + biguanide	66 (5.6)	39 (3.0)	–	105 (4.2)
Thiazolidinedione + sulfonylurea	2 (0.2)	1 (0.1)	–	3 (0.1)
Other	3 (0.3)	4 (0.3)	–	7 (0.3)
Patients ever given diet and exercise advice, *n* (%)	910 (77.3)	905 (68.8)	<0.0001	1,815 (72.8)
Patient with ≥1 hypoglycemic event (past 3 months), *n* (%)	61 (5.2)	58 (4.4)	NS	119 (4.8)
No. of hypoglycemic events among patients with ≥1 episode	8.2 ± 24.4	5.5 ± 9.5	–	6.8 ± 18.4
Diabetes complications, *n* (%)				
≥1 macrovascular complication	212 (18.0)	339 (25.8)	<0.0001	551 (22.1)
≥1 microvascular complication	173 (14.7)	281 (21.4)	<0.0001	454 (18.2)
Consultations to HCPs within the last 6 months				
Clinic visits to HCPs, *n* (%)	1,098 (93.3)	1,233 (93.8)	–	2,331 (93.5)
Phone calls to HCPs, *n* (%)	251 (21.3)	292 (22.2)	–	543 (21.8)
No. of clinic visits to HCPs	7.2 ± 7.0	7.7 ± 8.6	–	7.5 ± 7.9
No. of phone calls to HCPs	0.6 ± 1.4	0.72 ± 1.9	–	0.7 ± 1.7

Continuous data are means (SD). –, No statistical analysis performed

*b.i.d.* twice daily, *BMI* body mass index, *DPP*-*4* dipeptidyl peptidase-4, *GLP*-*1* glucagon-like peptide-1, *HbA*_*1c*_ glycated hemoglobin, *HCP* healthcare professionals, *HDL* high-density lipoprotein, *LDL* low-density lipoprotein, *NS* non-significant (using threshold for statistical significance of *P* < 0.05), *OAD* oral antidiabetic medication, *SMBG* self-monitoring of blood glucose

^a^Wilcoxon test used for continuous data. χ^2^ or Fisher’s exact tests used for categorical data

^b^Reported within the last 6 months prior to T1 (initiation)

^c^Comparisons under “Employment” and “Education” take into account all subcategories under these headings

^d^Cochrane–Armitage trend test for number of significant diagnoses (0, 1, 2, or more)

^e^Data used are those in only patients who used blood glucose monitoring

### Diabetes and Glucose Control

Overall, patients initiating exenatide b.i.d. reported a significantly shorter mean duration of diagnosed diabetes than those initiating insulin (8 ± 6 vs. 10 ± 7 years, respectively; *P* < 0.0001). This was consistent across all countries except Belgium (wherein the durations were similar) and was statistically significant in France (10 ± 6 vs. 13 ± 9 years; *P* < 0.01) and Greece (9 ± 6 vs. 12 ± 8 years; *P* < 0.0001; data not shown). Injectable therapy was initiated following an increase in HbA_1c_ during the previous 9–12 months, a trend consistent across countries. This increase in HbA_1c_ prior to initiation of injectable therapy was more pronounced in patients initiating insulin rather than exenatide b.i.d. At the point of initiation, patients who initiated exenatide b.i.d. had a significantly lower mean HbA_1c_ than those who initiated insulin (8.4% ± 1.4 vs. 9.2% ± 1.9; *P* < 0.0001). This was consistent across countries (Fig. 1b). Overall, 8.0% of patients (200/2,492) initiated injectable therapy despite having an HbA_1c_ measurement of <7% in the past 3 months. This percentage varied between 0% (Belgium) and 13.6% (Denmark) for exenatide b.i.d. (overall 10.7%) and between 0% (Denmark) and 6.4% (France) for insulin (overall 5.6%; data not shown). Patients who initiated exenatide b.i.d. also had significantly lower random blood glucose values than those who initiated insulin (Table 1). There were no discernible differences in patterns of OAD use at the initiation of injectable therapy between the cohorts (Table 1), with the possible exception that sulfonylurea use within the 12 months prior to inclusion in the study may have been higher in patients who initiated insulin than in those who initiated exenatide b.i.d. (although no statistical analysis was performed to confirm this). Overall, 5.2% of patients initiating exenatide b.i.d. and 4.4% of patients initiating insulin reported experiencing at least one hypoglycemic episode in the 3 months prior to baseline. Few patients reported severe or nighttime episodes (~5%) and there were no clear differences between cohorts.

Significantly fewer patients initiating exenatide b.i.d. had reported one or more macrovascular complication (18.0% vs. 25.8%; *P* < 0.0001) or microvascular complication (14.7% vs. 21.4%; *P* < 0.0001), compared with patients initiating insulin. Differences in the prevalence of macrovascular complications at the time of initiation of injectable therapy were particularly notable in Germany (15.5% vs. 24.2% in the exenatide b.i.d. and insulin cohorts, respectively; *P* < 0.01), Greece (21.4% vs. 28.2%; *P* < 0.05), and Sweden (14.6% vs. 29.3%; *P* < 0.05; data not shown). Differences in the frequency of microvascular complications were especially notable in France (11.7% vs. 22.3%; *P* < 0.05) and Greece (10.4% vs. 17.2%; *P* < 0.01).

### Comorbidity and Concomitant Medications

Patients initiating exenatide b.i.d. had a statistically higher incidence of comorbidities than patients initiating insulin (*P* = 0.0014), i.e., a diagnosis of hypertension (69.5% vs. 65.3%, respectively) and hyperlipidemia (54.5% vs. 48.7%, respectively). Overall, 85.5% of patients were using at least one concomitant (nondiabetes) medication (mainly cardiovascular, lipid-lowering, and antiplatelet agents) at initiation of injectable therapy and this was consistent across countries. There were no significant differences between cohorts in the use of cardiovascular, lipid-lowering, or antiplatelet agents (Table 1). Exenatide b.i.d. patients were statistically significantly more likely to have used weight-lowering medications than insulin patients (4.6% vs. 1.5%, respectively; *P* < 0.0001).

### Resource Use

Overall, 79.4% of patients self-monitored blood glucose, using a mean of 9.6 ± 8.3 test strips/week. There were no statistically significant differences between the cohorts in the number of contacts with healthcare professionals in the 6 months prior to baseline (Table 1).

### Factors Associated with Injectable Treatment Regimen and Propensity Score

A multiple logistic regression analysis (forward selection) identified BMI, age, and HbA_1c_ as the variables most strongly associated with a propensity for treatment assignment, with higher BMI, lower age, and lower HbA_1c_ indicating an increased probability of receiving exenatide b.i.d. (Table 2); [odds ratio (OR) exenatide b.i.d. vs. insulin: BMI (5 kg/m^2^ higher) 2.10, 95% CI 1.84–2.40; HbA_1c_ (1% higher): OR 0.77, 95% CI 0.69–0.86; age (5 years older) OR 0.82, 95% CI 0.76–0.88; all *P* < 0.0001). Other statistically significant factors associated with the treatment regimen were the “Disinhibited Eating Subscale” of the DHP-18 (which comprises five items that measure lack of eating control, response to food cues, and emotion [8]), random blood glucose, use of self-monitoring of blood glucose, receipt of diet/exercise advice, and low-density lipoprotein cholesterol (Table 2).

**Table 2:** Baseline variables that were statistically significantly associated with initiating exenatide b.i.d. compared with insulin (logistic regression using forward selection of variables significant in univariate analysis at threshold of *P* < 0.10)

Variable	OR	95% CI	*P* value
Body mass index			
1 kg/m^2^ higher	1.16	1.13–1.19	<0.0001
5 kg/m^2^ higher	2.10	1.84–2.40	<0.0001
Most recent HbA_1c_: 1% higher	0.77	0.69–0.86	<0.0001
Age			
1 year older	0.96	0.95–0.97	<0.0001
5 years older	0.82	0.76–0.88	<0.0001
10 years older	0.67	0.58–0.77	<0.0001
DHP-18 subscale: disinhibited eating (yes vs. no)	1.05	1.01–1.10	0.0083
Random blood glucose: 1 mmol/L higher	0.94	0.90–0.99	0.0141
Blood glucose self-monitoring			
1 test/week more	0.98	0.96–0.99	0.0107
5 tests/week more	0.88	0.81–0.96	0.0042
10 tests/week more	0.78	0.66–0.92	0.0042
Receipt of diet/exercise advice (yes vs. no)	1.67	1.13–2.46	0.0193
LDL cholesterol: 1 mmol/L higher	0.83	0.72–0.96	0.0138

*CI* confidence interval, *b.i.d.* twice daily, *DHP*-*18* Diabetes Health Profile-18, *HbA*_*1c*_ glycated hemoglobin, *LDL* low-density lipoprotein, *OR* odds ratio exenatide b.i.d. versus insulin (OR values >1 indicate variables that were associated with increased probability of receiving exenatide b.i.d., OR values <1 indicate variables associated with decreased probability of receiving exenatide b.i.d.)

A propensity score analysis based on baseline demographic and clinical variables underlined the differences between the treatment cohorts. The mean propensity score value for the probability of receiving exenatide b.i.d. was 0.63 ± 0.23, and of receiving insulin was 0.33 ± 0.23 (Table 3). Evaluation of propensity score quintiles indicated that 51% of patients could be matched (1,278 patients, 639 per cohort), largely representing the upper tail of the exenatide b.i.d. distribution and the lower tail of the insulin distribution.

**Table 3:** Overall propensity scores and distribution by quintile for each cohort

	Exenatide b.i.d. (*n* = 1,177)		Insulin (*n* = 1,315)	
Mean score:	0.63 ± 0.23		0.33 ± 0.23	
Quintile	*n* (%)	Mean score	*n* (%)	Mean score
1	42 (3.6)	0.13 ± 0.04	456 (34.7)	0.11 ± 0.05
2	145 (12.3)	0.29 ± 0.05	353 (26.8)	0.27 ± 0.05
3	241 (20.5)	0.46 ± 0.05	258 (19.6)	0.45 ± 0.05
4	327 (27.8)	0.65 ± 0.06	171 (13.0)	0.64 ± 0.05
5	422 (35.9)	0.87 ± 0.07	77 (5.9)	0.84 ± 0.06

Quintiles calculated over both cohorts combined

## Discussion

Well-designed, observational (“real-world”) studies are essential to enhancing the evidence upon which the management of T2DM is based [5]. The CHOICE study, a large prospective observational study conducted in six European countries, has provided the first available data on the way exenatide b.i.d. is used in clinical practice across Europe. The present report focuses on the baseline characteristics of patients and it identifies differences between patients who initiate exenatide b.i.d. and those who initiate starter insulin at the discretion of the treating physician. As well as being of intrinsic interest, these data will also have implications for the comparability of clinical outcomes between the CHOICE cohorts in future publications, and perhaps for the comparability between exenatide b.i.d. and insulin data from clinical practice more generally.

Overall, patients initiating exenatide b.i.d. were characterized by: younger age; higher body weight, BMI, and waist circumference; higher diastolic blood pressure, lower total and LDL cholesterol levels; shorter time since diabetes diagnosis; and better glycemic control. A lower proportion of patients initiating exenatide b.i.d. had microvascular and macrovascular complications (compared with patients initiating insulin), a finding that might reflect their younger mean age, glycemic control (HbA_1c_), and duration of diabetes. The differences between the two cohorts in key variables such as age, obesity measures, and HbA_1c_ were consistent across the participating countries, although the statistical significance of some inter-cohort differences was limited by the sample size in individual countries. Overall BMI, HbA_1c_, and age were factors significantly associated with differentiation between exenatide b.i.d. and insulin (*P* < 0.0001).

The risk of treatment-induced hypoglycemia is an important consideration during treatment selection [1–3], especially in patients with an HbA_1c_ close to or within target levels; the recently published American Diabetes Association (ADA)/European Association for the Study of Diabetes (EASD) consensus statement for T2DM management specifies exenatide b.i.d. as an option when hypoglycemia is a particularly important consideration [7]. Consistent with this proposed treatment algorithm, the frequency of recent hypoglycemia in CHOICE was numerically higher in patients who initiated exenatide b.i.d. than in the insulin cohort, but the number of affected patients was small and the difference was not statistically significant.

The finding that exenatide b.i.d. was favored in patients with higher body weight is not surprising. Patients treated with exenatide b.i.d. show significant weight loss compared with those treated with insulin [1–3]. The ADA/EASD consensus statement and the American Association of Clinical Endocrinologists treatment algorithm identify the use of exenatide b.i.d. as an option when weight loss is a major consideration [7, 12].

The mean HbA_1c_ level in both cohorts in CHOICE exceeded the recommended target level of <7% [7] prior to the initiation of injectable therapy. Mean HbA_1c_ levels rose gradually during the 18–24 months prior to baseline. A steeper rise occurred in the 6 months prior to baseline and this appeared more marked in the insulin cohort (reaching 9.2%) than in the exenatide b.i.d. cohort (reaching 8.4%). Although this finding may reflect different disease progression in the two cohorts prior to the initiation of either exenatide b.i.d. or insulin, missing pre-baseline HbA_1c_ records in some patients make interpretation of this phenomenon difficult. These findings are consistent with previous observational evidence that insulin initiation is very often delayed for years despite poor glycemic control on oral glucose-lowering medications [9, 13–15].

The finding that patients initiating exenatide b.i.d. in CHOICE had lower HbA_1c_ levels than those initiating insulin is consistent with US observational data [16] and with the ADA/EASD consensus statement that identifies exenatide b.i.d. as an option for patients with glycemic control closer to target levels [7]. However, clinical data support use of exenatide b.i.d. at various ranges of HbA_1c_ including high values (>9%) and not only where HbA_1c_ is close to target levels [17–19]. The consistently higher baseline blood glucose values observed among insulin patients, coupled with a longer time since diagnosis, may indicate that exenatide b.i.d. is used earlier in the disease to intensify therapy and thereby delay the need for insulin initiation. A minority of patients initiated insulin (10.7%) or exenatide b.i.d. (5.6%) despite having a baseline HbA_1c_ of <7%.

The observation (on univariate analysis) of a higher proportion of diagnosed dyslipidemia or hypertension inpatients initiating exenatide b.i.d. is of unclear significance but may be related to body weight. Similar findings were observed in the aforementioned retrospective database analysis in the United States [16]. In common with CHOICE, this previous analysis also found that patients initiating exenatide b.i.d. had significantly lower rates of macrovascular and microvascular complications than those initiating insulin [16]. Overall, the mean blood pressure values among CHOICE patients at baseline would classify the population at low risk according to the target of 130/80 mmHg recommended by the International Diabetes Federation [20], and a Task Force of the European Society of Cardiology and the EASD [21]. Even if blood pressure values were elevated, hypertension is unlikely to be a driver of treatment choice independently of HbA_1c_ and weight. Patients who initiated insulin had significantly higher creatinine levels than those who initiated exenatide b.i.d. This could reflect the higher mean age in the insulin cohort. It is also possible that renal complications, as well as other complications, might have contributed to the initiation of insulin. The lack of data on glomerular filtration rate hampers interpretation of the creatinine data.

Univariate analyses showed that patients receiving exenatide b.i.d. were more likely than insulin recipients to be employed. Primary occupational status was correlated with age, and only age showed a significant association with treatment selection in the multivariate analysis. The higher educational status in the exenatide b.i.d. cohort compared with the insulin cohort on univariate analysis may reflect the possibility that more educated patients are more likely to ask for newer treatments, but this is speculation. Educational status was not included in the multivariate analysis owing to a relatively high proportion of missing data.

The CHOICE study has several limitations. While the data allow a multivariate analysis to help improve the understanding of the measured variables most strongly associated with the choice of injectable therapy, other variables that were not captured, such as clinical guidelines and patient preference, may be at least as clinically relevant. As significant differences between the cohorts are present, outcome results observed in these groups are not directly comparable. The matched population refers to those patients in whom outcomes can be statistically compared. However, this largely represents the upper tail of the exenatide b.i.d. propensity score distribution and the lower tail of the insulin distribution. Therefore, caution is advised when interpreting comparative outcomes. Although CHOICE was designed to recruit a representative sample of patients, the degree to which the data can be generalized is unclear for several reasons. Firstly, patients were mostly recruited in secondary-care centers and hence the data may not be generalizable to the primary-care setting. Secondly, the similarity in the size of the exenatide b.i.d. and insulin cohorts does not reflect the ratio in routine clinical practice, wherein many more patients initiate insulin than exenatide b.i.d. The CHOICE population appears similar to that of the observational “Insulin titration; gaining an understanding of type 2 diabetes in Europe” (INSTIGATE) study [9], which looked at T2DM patients initiating insulin therapy, in terms of such variables as mean age, BMI, and duration of diabetes among participants, although INSTIGATE patients had a higher mean HbA_1c_ at the initiation of insulin (9.6% vs. 9.2% in CHOICE). The small sample size in some countries reduces the statistical power of the inter-cohort analysis. The variation in sample sizes between the six countries also limits the robustness of inter-country comparisons, although the inter-cohort comparisons nevertheless showed a considerable degree of international consistency. There was also variation between countries in the ratios of exenatide b.i.d. and insulin patients.

In conclusion, this analysis has improved understanding of which patients are initiated on exenatide b.i.d. or insulin in routine clinical practice. The cohort of patients who initiated exenatide b.i.d. in CHOICE was younger, more obese, and had a lower HbA_1c_ than those who initiated insulin. These differences appear to reflect the recommendations for the use of these two therapies, with exenatide b.i.d. identified as an option when weight gain is a particular concern and when HbA_1c_ is modestly raised [7, 12]. The data suggest that the patient profile may contribute to the prescribing of injectable glucose-lowering therapy regimen for patients with T2DM.

## References

[1] Heine RJ, Van Gaal LF, Johns D (2005). Exenatide versus insulin glargine in patients with suboptimally controlled type 2 diabetes: a randomized trial. Ann Intern Med.

[2] Barnett AH, Burger J, Johns D (2007). Tolerability and efficacy of exenatide and titrated insulin glargine in adult patients with type 2 diabetes previously uncontrolled with metformin or a sulfonylurea: a multinational, randomized, open-label, two-period, crossover noninferiority trial. Clin Ther.

[3] Nauck MA, Duran S, Kim D, Johns D, Northrup J, Festa A (2007). A comparison of twice-daily exenatide and biphasic insulin aspart in patients with type 2 diabetes who were suboptimally controlled with sulfonylurea and metformin: a non-inferiority study. Diabetologia.

[4] Klonoff DC, Buse JB, Nielsen LL, Guan X, Bowlus CL, Holcombe JH (2008). Exenatide effects on diabetes, obesity, cardiovascular risk factors and hepatic biomarkers in patients with type 2 diabetes treated for at least 3 years. Curr Med Res Opin.

[5] Ligthelm RJ, Borzì V, Gumprecht J, Kawamori R, Wenying Y, Valensi P (2007). Importance of observational studies in clinical practice. Clin Ther.

[6] Mann CJ (2003). Observational research methods. Research design II: cohort, cross sectional, and case-control studies. Emerg Med J.

[7] Nathan DM, Buse JB, Davidson MB (2009). Medical management of hyperglycemia in type 2 diabetes: a consensus algorithm for the initiation and adjustment of therapy. A consensus statement of the American Diabetes Association and the European Association for the Study of Diabetes. Diabetes Care.

[8] Meadows K, Abrams S, Sandback A (2000). Adaptation of the diabetes health profile (DHP-1) for use with patients with type 2 diabetes mellitus: psychometric evaluation and cross-cultural comparison. Diabet Med.

[9] Jones S, Benroubi M, Castell C (2009). Characteristics of patients with type 2 diabetes mellitus initiating insulin therapy: baseline data from the INSTIGATE study. Curr Med Res Opin.

[10] PHARMO report: treatment pathways of type 2 DM patients starting insulin regimens. Utrecht, The Netherlands, January 2007 (Lilly, data on file).

[11] Yue LQ (2007). Statistical and regulatory issues with the application of propensity score analysis to nonrandomized medical device clinical studies. J Biopharm Stat.

[12] Rodbard HW, Jellinger PS, Davidson JA (2009). Statement by an American Association of Clinical Endocrinologists/American College of Endocrinology consensus panel on type 2 diabetes mellitus: an algorithm for glycemic control. Endocr Pract.

[13] Nichols GA, Koo YH, Shah SN (2007). Delay of insulin addition to oral combination therapy despite inadequate glycemic control: delay of insulin therapy. J Gen Intern Med.

[14] Rubino A, McQuay LJ, Gough SC, Kvasz M, Tennis P (2007). Delayed initiation of subcutaneous insulin therapy after failure of oral glucose-lowering agents in patients with type 2 diabetes: a population-based analysis in the UK. Diabet Med.

[15] Brown JB, Nichols GA, Perry A (2004). The burden of treatment failure in type 2 diabetes. Diabetes Care.

[16] Fabunmi R, Nielsen LL, Quimbo R (2009). Patient characteristics, drug adherence patterns, and hypoglycemia costs for patients with type 2 diabetes mellitus newly initiated on exenatide or insulin glargine. Curr Med Res Opin.

[17] Buse JB, Henry RR, Han J (2004). Effects of exenatide on glycemic control over 30 weeks in sulfonylurea-treated patients with type 2 diabetes. Diabetes Care.

[18] Kendall DM, Riddle MC, Rosenstock J (2005). Effects of exenatide (exendin-4) on glycemic control over 30 weeks in patients with type 2 diabetes treated with metformin and a sulfonylurea. Diabetes Care.

[19] Buysschaert M, Preumont V, Oriot PR (2010). One year metabolic outcomes in patients with type 2 diabetes treated with exenatide in routine practice. Diabetes Metab.

[20] IDF Clinical Guidelines Task Force. Global guideline for type 2 diabetes. Brussels: International Diabetes Federation, 2005.

[21] Rydén L, Standl E, Bartnik M (2007). Guidelines on diabetes, pre-diabetes, and cardiovascular diseases: executive summary. The Task Force on Diabetes and Cardiovascular Diseases of the European Society of Cardiology (ESC) and of the European Association for the Study of Diabetes (EASD). Eur Heart J.

